# Regulation of Plant Microprocessor Function in Shaping microRNA Landscape

**DOI:** 10.3389/fpls.2018.00753

**Published:** 2018-06-05

**Authors:** Jakub Dolata, Michał Taube, Mateusz Bajczyk, Artur Jarmolowski, Zofia Szweykowska-Kulinska, Dawid Bielewicz

**Affiliations:** Department of Gene Expression, Institute of Molecular Biology and Biotechnology, Faculty of Biology, Adam Mickiewicz University in Poznan, Poznan, Poland

**Keywords:** microprocessor, DCL1, SE, HYL1, miRNA biogenesis, *Arabidopsis*

## Abstract

MicroRNAs are small molecules (∼21 nucleotides long) that are key regulators of gene expression. They originate from long stem–loop RNAs as a product of cleavage by a protein complex called Microprocessor. The core components of the plant Microprocessor are the RNase type III enzyme Dicer-Like 1 (DCL1), the zinc finger protein Serrate (SE), and the double-stranded RNA binding protein Hyponastic Leaves 1 (HYL1). Microprocessor assembly and its processing of microRNA precursors have been reported to occur in discrete nuclear bodies called Dicing bodies. The accessibility of and modifications to Microprocessor components affect microRNA levels and may have dramatic consequences in plant development. Currently, numerous lines of evidence indicate that plant Microprocessor activity is tightly regulated. The cellular localization of HYL1 is dependent on a specific KETCH1 importin, and the E3 ubiquitin ligase COP1 indirectly protects HYL1 from degradation in a light-dependent manner. Furthermore, proper localization of HYL1 in Dicing bodies is regulated by MOS2. On the other hand, the Dicing body localization of DCL1 is regulated by NOT2b, which also interacts with SE in the nucleus. Post-translational modifications are substantial factors that contribute to protein functional diversity and provide a fine-tuning system for the regulation of protein activity. The phosphorylation status of HYL1 is crucial for its activity/stability and is a result of the interplay between kinases (MPK3 and SnRK2) and phosphatases (CPL1 and PP4). Additionally, MPK3 and SnRK2 are known to phosphorylate SE. Several other proteins (e.g., TGH, CDF2, SIC, and RCF3) that interact with Microprocessor have been found to influence its RNA-binding and processing activities. In this minireview, recent findings on the various modes of Microprocessor activity regulation are discussed.

## Introduction

Mature microRNAs are derived from long primary transcripts (pri-miRNAs) that are produced by RNA Polymerase II (RNAPII). They are capped at their 5′ ends and possess a poly A tail at their 3′ ends (Xie et al., 2005). Pri-miRNA levels are tightly regulated at the transcriptional (Zhang et al., 2013; Sun et al., 2015, 2018), co-transcriptional (Fang et al., 2015; Dolata et al., 2016) and post-transcriptional levels (Ben Chaabane et al., 2013; Bielewicz et al., 2013; Zhang et al., 2015; Barciszewska-Pacak et al., 2016; Knop et al., 2017; Stepien et al., 2017; Yu et al., 2017). Interestingly, in many cases, changes in the level of a given pri-miRNA are not reflected in changes in the level of the mature microRNA (Barciszewska-Pacak et al., 2015; Dolata et al., 2016). This might be a consequence of regulation at the pri-miRNA or pre-miRNA (intermediate product during microRNA biogenesis) processing/degradation step. Production of microRNAs is driven by a complex called Microprocessor, which in *Arabidopsis* consists of three core proteins: the RNase type III enzyme Dicer-Like 1 (DCL1), the zinc finger protein Serrate (SE), and the double-stranded RNA binding protein Hyponastic Leaves 1 (HYL1). Microprocessor term was originally coined for a nuclear protein complex in animal cells for pre-miRNA production (Denli et al., 2004; Gregory et al., 2004).

## Microprocessor Components Localization

In plants, Microprocessor action is limited to the nucleus (Fang and Spector, 2007; Yu et al., 2017), whereas localization of its components is not restricted to one cellular compartment. According to current knowledge, the first steps of plant microRNA biogenesis occur in specialized nuclear foci called dicing bodies (D-bodies) (Fang and Spector, 2007). Together, DCL1 and HYL1 in the nucleus are found almost exclusively in D-bodies. However, SE is present in D-bodies as well as in nuclear speckles that contain serine/arginine-rich (SR) splicing factors (Ali et al., 2003). How the Microprocessor complex is assembled in D-bodies and how it is recruited to pri-miRNAs are still not clear. Nevertheless, several factors have been shown to be important for D-body formation and effective cleavage of microRNA precursors.

A growing amount of evidence indicates a direct link between RNAPII transcription and the biogenesis of small RNAs in *Arabidopsis* (Fang et al., 2015; Dolata et al., 2016; Liu et al., 2017). One of the overlapping elements is Elongator complex, firstly described in yeast (Otero et al., 1999) and further purified from plant cells. Elongator was described as a six-component complex involved in the regulation of transcription elongation (Nelissen et al., 2010). Fang et al. (2015) have shown that disruption of the Elongator complex results in reduced RNAPII occupancy at tested *MIR* genes and lower levels of a few pri-miRNAs. Mutants of two Elongator subunits (*elp2-2* and *elp5-1*) have disrupted DCL1 localization and a reduced number of D-bodies. Furthermore, all core Microprocessor components interact with Elongator complex subunits (ELP2, ELP4, and ELP5-Elongator complex Proteins 2/4/5). DCL1 associates with chromatin on *MIR* loci, and a functional Elongator complex is necessary for DCL1 recruitment to nascent *MIR* transcripts. These data suggest that the processing of at least some pri-miRNAs occurs co-transcriptionally.

More evidence for a connection between transcription and Microprocessor was presented by Wang et al. (2013), who described two *Arabidopsis* NOT2 (Negative on TATA-less 2) proteins (NOT2a and NOT2b) as factors that promote microRNA production. In yeasts, NOT2 was shown to bind directly to RNAPII and to promote transcription elongation (Kruk et al., 2011). Similarly, in *Arabidopsis*, NOT2b co-precipitates with the large subunit of RNAPII and affects transcription. Moreover, NOT2b interacts with DCL1 and SE; however, it does not interact with HYL1. Furthermore, a *not2a-1 not2b-1* double mutant was shown to have an increased number of nuclear foci containing DCL1. Still, the possibility that NOT2 links *MIR* gene transcription and post-transcriptional processing for better coordination and efficiency cannot be excluded.

In *Arabidopsis*, the MOS4-Associated Complex (MAC) (Palma et al., 2007; Monaghan et al., 2009) is a counterpart of the NineTeen Complex (NTC) in yeast (Fabrizio et al., 2009) and is directly linked to transcription and microRNA biogenesis (Zhang et al., 2013, 2014). Recently, it was found that mutants of MAC subunits (*mac7-1* and double *mac3a mac3b*) have reduced number of HYL1-containing *D-bodies* (Jia et al., 2017; Li et al., 2018). Authors suggest that it is a consequence of the fact that Microprocessor complex assembly requires pri-miRNA (Wu et al., 2013). Therefore decreased level of pri-miRNAs in *mac* mutants may affect HYL1 localization.

These assumptions are made based on the previous paper by Wu et al. (2013) who found the RNA-binding protein MOS2 (Modifier of Snc1) is supporting D-body assembly. In a *mos2-2* mutant, HYL1 localization was found to be different than that in WT plants as it was relatively homogeneous in the nucleus; however, HYL1 interactions with DCL1 and SE were not affected. MOS2 does not interact directly with core Microprocessor components but instead binds pri-miRNAs. Additionally, in the absence of MOS2, the association of HYL1 with pri-miRNAs is significantly reduced (Zhang et al., 2005). These data suggest that microRNA precursors may serve as scaffolds for D-body formation.

The balance between pri-miRNA and Microprocessor components assembly may be disturbed by over-accumulation of pre-mRNA splicing intermediates. Non-debranched intron lariats sequestrate dicing complexes and negatively affect pri-miRNA processing. *Arabidopsis* mutant in debranching enzyme (*dbr1-2*) shows increased number of DCL1 and HYL1 nuclear bodies (Li et al., 2016).

The distribution of HYL1-GFP indicates that HYL1 is present in the nucleus and the cytoplasm (Han et al., 2004). HYL1 degradation in the cytoplasm is regulated by the RING-finger E3 ligase COP1 (Constitutive Photomorphogenic 1) (Cho et al., 2014). During the day, COP1 moves to the cytoplasm and indirectly protects HYL1 from degradation, most likely by inhibiting an undefined protease. During the night, COP1 remobilizes to the nucleus, allowing the protease to cleave the N-terminus of HYL1. This specific cleavage inhibits HYL1 function and causes an immediate reduction in correctly processed microRNAs (Cho et al., 2014).

A connection between light signaling and microRNA biogenesis comes from studies on Phytochrome Interacting Factor 4 (PIF4). It was shown that PIF4 promotes the destabilization of both: DCL1 and HYL1 during dark-to-red-light transition (Sun et al., 2018).

Processing of *MIR* gene transcripts occurs in the nucleus, and effective cytoplasm–nucleus trafficking of Microprocessor components is necessary for its proper function. Recently, KETCH1 (Karyopherin Enabling the Transport of the Cytoplasmic HYL1) was described as an HYL1-interacting importin-β protein (Zhang et al., 2017). *KETCH1* null mutants are embryo-lethal, whereas the downregulation of KETCH1 using artificial microRNAs causes a reduction in HYL1 level in the nucleus, although SE localization in the nucleus is not affected. A decreased level of KETCH1 leads to disturbances in microRNA production (accumulation of several pri-miRNAs as well as pre-miRNAs and reduced levels of mature microRNAs). An *amiR*-*ketch1* and *hyl1-2* double mutant was found to resemble the *hyl1-2* phenotype morphologically as well as at the microRNA level, which indicates that both proteins act in the same pathway. It is not known if KETCH1 functions are limited to HYL1 cytoplasm–nucleus transport; however, the regulation of HYL1 level and its accessibility in different tissues and under different growth conditions might play an important role in the regulation of proper levels of miRNAs that are HYL1-dependent (Szarzynska et al., 2009).

## Post-Translational Modifications (PTMs) of Microprocessor Components

Most proteins require PTMs for proper function. More than 40 different post-translational protein modifications have been identified (Beck-Sickinger and Mörl, 2006), and they can play important roles in protein folding, subcellular localization, catalytic activity, or stability. For the plant Microprocessor, the phosphorylation of HYL1 is the only PTM that has been found to be crucial for efficient microRNA production. The interplay between protein phosphorylation and dephosphorylation is known to enable the rapid and efficient tuning of protein function. Using a forward genetic screen, Manavella et al. (2012) found that a mutation in the *CPL1* (C-Terminal Domain Phosphatase-like 1) gene causes impaired processing of microRNA precursors and aberrant strand selection during RISC loading. CPL1 encodes a phosphatase that was shown to dephosphorylate the C-terminal domain (CTD) of the RNAPII largest subunit specifically at Ser5 (Koiwa et al., 2004). *In vivo*, CPL1 interacts with two components of the plant Microprocessor, SE and HYL1 (Jeong et al., 2013). Two serine residues of HYL1, S42 and S159, are especially important for HYL1 function, and hyperphosphorylated HYL1 is inactive (Manavella et al., 2012). Both serine residues are located within the dsRNA binding domains of HYL1. Dephosphorylation of HYL1 by CPL1 is stimulated by a protein called RCF3 (Regulator of CBF Gene Expression 3, also known as HOS5 or SHINY1 (Jiang et al., 2013; Chen et al., 2015; Karlsson et al., 2015). *RCF3* expression is restricted to the vegetative shoot apical meristem, young leaf primordia and newly emerging leaves, which suggests that fine-tuning of HYL1 activity via phosphorylation can be tissue specific (Karlsson et al., 2015). Moreover, the expression of *RCF3* is reduced by salt, hyperosmotic stress, and ABA. This may indicate that plants modulate the phosphorylation status of HYL1 in response to environmental changes (Jiang et al., 2013). Another protein that dephosphorylates HYL1 is PP4 (Protein Phosphatase 4, also termed PPX) (Su et al., 2017). PP4 is a highly conserved protein among eukaryotes that functions to assist specific regulatory subunits (for example, SMEK1 in plants, PP4RS in mammals, and PSY2 in yeast (Gingras et al., 2005; Kataya et al., 2017; Su et al., 2017). In *Arabidopsis thaliana*, the PP4 phosphatase is encoded by two genes (PP4-1 and PP4-2), the proteins of which share 93% sequence identity and have the same expression pattern, suggesting that their biological functions might be very similar if not redundant (Pujol et al., 2000). Attempts to obtain stable *A. thaliana* knockdown/out lines for the PP4-1/2 genes have been unsuccessful; however, knockout mutants of the PP4 regulatory subunit SMEK1 (Suppressor of MEK1) are viable (Kataya et al., 2017; Su et al., 2017). In *smek1* mutants, microRNA expression levels are reduced due to the accelerated degradation of hyperphosphorylated HYL1. Importantly, SMEK1 protects HYL1 from degradation in a COP1- and light-independent manner; therefore, the regulation of HYL1 activity by PP4 represents another regulatory network present in plants (Su et al., 2017).

**FIGURE 1:**
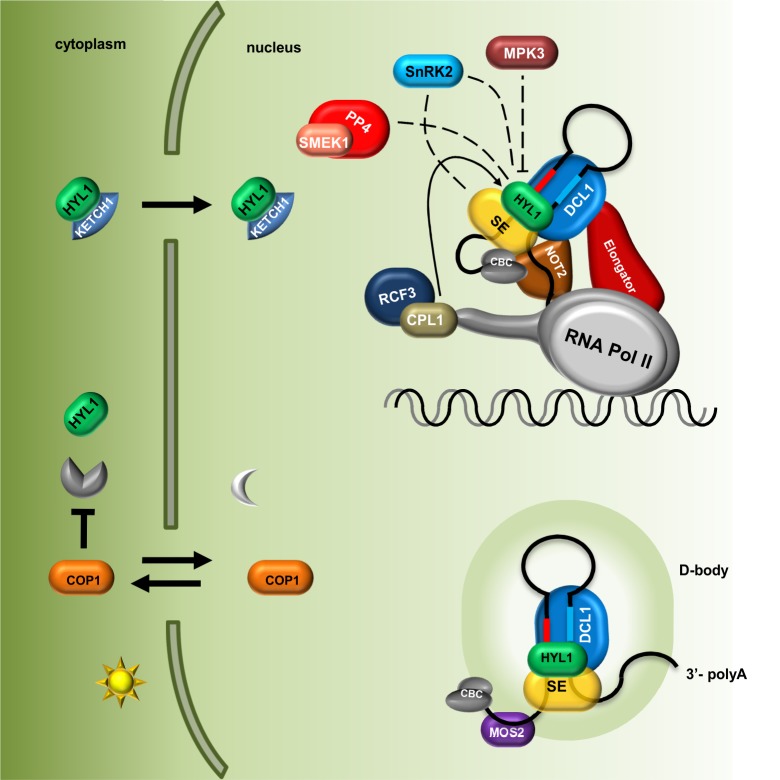
Plant microRNA biogenesis is fine-tuned via regulation of Microprocessor components localization and their post-translational modifications. The level of HYL1 in the cytoplasm is indirectly regulated by COP1 in a light-dependent manner; HYL1 nuclear import is mediated by KETCH1 importin. Core components of the Microprocessor: DCL1, SE and HYL1 as well as microRNA precursors are located and interact with each other in the D-bodies. MOS2 is important for the D-bodies assembly however, does not interact directly with the Microprocessor. The Elongator complex and NOT2 proteins form a bridge coupling transcription and processing of microRNAs. Phosphorylation status of HYL1 is crucial for its efficient function and is a consequence of an interplay between kinases (MPK3 and SNRK2) and phosphatases (CPL1 and PP4). Direct influence of SnRK2 on SE and HYL activity is not known. CBC, nuclear Cap Binding Complex; dashed lines indicate that exact localization of the interactions is unknown.

Beside phosphatases, kinases have also been found to be important for HYL1 phospho-regulation. HYL1 is phosphorylated by MPK3 (Mitogen-Activated Protein Kinase 3) and SnRK2 (SNF1-related protein kinase subfamily 2) (Raghuram et al., 2015; Yan et al., 2017). In *mpk3* mutant plants, HYL1 protein accumulates, and consequently, the levels of mature microRNAs are significantly higher than those in wild-type plants. Interestingly, the repertoires of small RNAs are affected in both *mpk3* and *cpl1* mutants, but they do not overlap with each other, which may suggest that CPL1 (phosphatase) and MPK3 (kinase) act separately in parallel pathways (Raghuram et al., 2015). Furthermore, upon ABA treatment, MPK3 activation depends on the presence of HYL1 in the cell, which suggests that MPK3 and HYL1 are regulated in a feedback loop (Lu et al., 2002). The SnRK2 subfamily consists of 10 members (SnRK2.1-10) among which SnRK2.2/2.3/2.6 are involved in pri-miRNA processing and are strongly activated by ABA treatment. Surprisingly, in a *snrk2.2/3/6* triple mutant, the levels of HYL1 and mature microRNAs were found to be decreased (Yan et al., 2017). Yan et al. (2017) have shown that SnRK2.6 phosphorylates HYL1 *in vitro* and that SnRK2.2, SnRK2.3, and SnRK2.6 interact with HYL1 in plants. Many putative phosphorylation sites were found in the HYL1 protein (Supplementary Table S1); however, the precise amino acid residues that are phosphorylated in HYL1 by MPK3 and SnRK2.2/2.3/2.6 have not been identified. Thus, various HYL1 phosphorylation patterns might exert different functional effects on pri-miRNA biogenesis. Yan et al. (2017) showed that SnRK2.6 can phosphorylate the SE protein *in vitro* in addition to HYL1. The observation that SE can be phosphorylated in plants was reported previously (see chapter below) (Wang et al., 2013), but, currently, nothing is known about the effect of this modification on SE localization, stabilization, or activity. A model presenting current knowledge on the role of PTMs in Microprocessor activity regulation and localization of its components is shown in the **Figure 1**.

## Structural Aspects of PTMs of Core Microprocessor Complex Proteins

**FIGURE 2:**
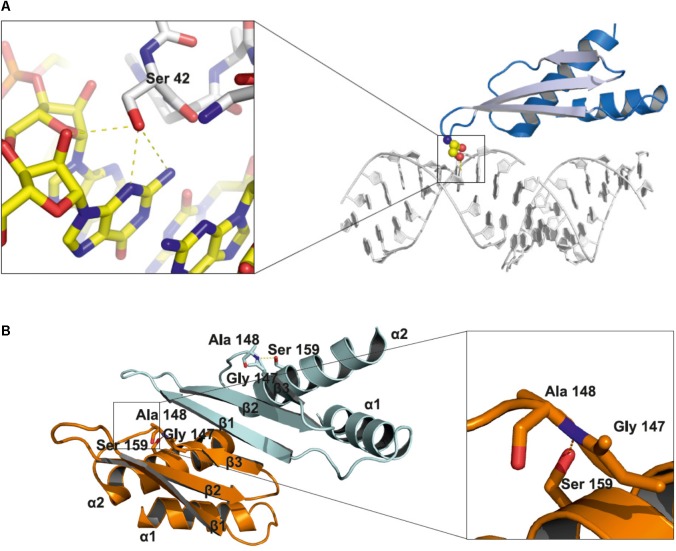
Structural insights into the role of serine 42 and serine 159 residues in the HYL1 protein based on the crystal structure from Yang et al. (2010). **(A)** Interactions of non-phosphorylated serine 42 with minor groove of dsRNA. **(B)** Stabilization of beta strand 3 by the hydrogen bond between non-phosphorylated serine 159 and peptide bond between glycine 147 and alanine 148.

DCL1 is the largest protein in the Microprocessor complex. It contains several domains: a helicase domain at the N-terminus, a domain of unknown function 283, a PAZ domain, two catalytic RNase III domains and two dsRNA binding domains at the C-terminus. The HYL1 protein contains two dsRNA-binding domains (dsRBD1 and dsRBD2) at the N-terminus and six 28-amino acid imperfect repeats at the C-terminus. The SERRATE protein possesses a core domain (195–543) that can be divided into three regions: an N-terminal alpha helical fragment, a middle domain fragment and a C2H2 zinc finger fragment. Both the N and C termini of SERRATE are predicted to be disordered in solution (Machida et al., 2011). The structures of the core domains of the *A. thaliana* SERRATE and HYL1 proteins and the second dsRBD domain of DCL1 have been determined (Rasia et al., 2010; Yang et al., 2010; Machida et al., 2011; Burdisso et al., 2012, 2014). Moreover, the structure of the dsRBD1 of HYL1 was solved as a complex containing a short 10-bp RNA duplex. Both dsRBD domains of HYL1 possess an alpha-beta-beta-beta-alpha fold, which is a signature of the dsRBD domain family (Masliah et al., 2013). Thus far, no data regarding how PTMs might interfere with the structure of Microprocessor proteins have been reported. Serine 42 is located within the loop between beta strand 1 and beta strand 2 of the HYL1 dsRBD1. Using the published structure, we noticed that the side chain of serine 42 may interact with the minor groove of the dsRNA and may form hydrogen bonds with the N2 and N3 nitrogen atoms of guanine and the 2′ hydroxyl group of the ribose ring (**Figure 2A**). A bulky phosphate group attached to serine 42 could potentially interfere with the minor groove interactions and negatively regulate binding to RNA duplexes. Serine 159 is localized in alpha helix 2 of the HYL1 dsRBD2, and it may interact with the loop between beta strand 3 and alpha helix 2. In addition, this serine may form a hydrogen bond with the nitrogen from the peptide bond between glycine 147 and alanine 148 in the opposite loop of the same HYL1 molecule (**Figure 2B**). Yang et al. (2014) found that a G147E mutation significantly reduces dimerization of the HYL1 protein. Moreover, this substitution was present in a *hyl1-3* mutant (Manavella et al., 2012). Therefore, serine 159 phosphorylation could destabilize the hydrogen bond network, leading to the mislocation of beta strand 3, the disruption of the dimerization interface and, ultimately, the inactivation of HYL1.

From high-throughput studies of the *A. thaliana* phospho-proteome, several phosphorylation sites in the SERRATE protein were identified and deposited in the PhosPhAt 4.0 database (Durek et al., 2010). The phosphorylated residues are mostly located within the N-terminal region, a low complexity domain rich in proline and serine residues, and within the middle fragment from the core structure domain. In addition, one phosphorylation site was found within the C-terminal fragment. A list of the phosphorylation sites found in the SERRATE and HYL1 proteins is shown in Supplementary Table S1. The N-terminal fragment of SERRATE (amino acids 1–194) is predicted to be disordered in solution. Additionally, the amino acid sequence conservation in this region is relatively low in comparison to that of the core domain (195–543). Nevertheless, the overall amino acid composition between SERRATE proteins from different species is very similar. Phosphorylation of low complexity domains has been shown to affect aggregation, local structure and protein-protein interactions (Kwon et al., 2013; Monahan et al., 2017). The N-terminal domain of SERRATE is responsible for its interaction with the two U1 small nuclear ribonucleoprotein particle (U1 snRNP) proteins, PRP40b and PRP40a, and deletion of the N-terminal domain causes more homogeneous nuclear localization in comparison to the speckle-like localization of the wild-type protein (Knop et al., 2017). Thus, the phosphorylation status of this domain in SERRATE may affect interactions with a large number of interacting partners in different processes in which SE is involved.

## Negative Feedback Regulation of Microprocessor

To provide fine tuning of microRNA production and to maintain balance in mRNA target degradation, Microprocessor components are regulated at the post-transcriptional level by a negative feedback loop. This feedback regulation of Microprocessor was first shown in 2003 by Carrington group. Xie et al. (2003) showed that DCL1 mRNA level is regulated by miRNA162. In wild-type plants, the *DCL1* transcript is in relatively low-abundance because functional DCL1 catalyzes miRNA production, and miRNA162 targets the *DCL1* transcript for degradation. However, in mutants with impaired miRNA biogenesis (for example *dcl1-7, hen1-1*), increased level of *DCL1* mRNA has been detected. Moreover, plants expressing the P1/HC-pro protein [Turnip mosaic virus (TuMV) RNA silencing suppressor], which inhibits the small RNA-guided cleavage of RNAs, had increased DCL1 mRNA level (Xie et al., 2003). The abundance of the *DCL1* transcript is also regulated by the production of miRNA838, which is encoded within the 14th intron of the DCL1 pre-mRNA. The generation of this miRNA is a consequence of DCL1 pre-mRNA cleavage into two non-functional transcripts that are 4- and 2.5-kb in length (Rajagopalan et al., 2006). Rajagopalan et al. (2006) suggested that higher level of DCL1 protein results in more efficient processing of DCL1 primary transcripts by Microprocessor than its recognition by the Spliceosome, which results in a higher level of miR838 and a lower level of *DCL1* transcript in the cell. Similar to DCL1, the SE level is determined by a negative feedback loop that involves miR863-3p. This microRNA targets the 3′ UTR of SE mRNA as well as two negative regulators of plant defense: ARLPK1 and ARPLK2. The level of miR863-3p increases after bacterial infection and silences two negative regulators of plant defense by cleaving their mRNAs. At subsequent steps of infection, when at its highest level, miR863-3p inhibits the translation of SE mRNA, which results in lower efficiency of miRNA biogenesis and decreased miRNA levels, including those of miR863-3p (Niu et al., 2016).

## Author Contributions

JD, MT, MB, AJ, ZS-K, and DB participated in preparation of draft manuscript. MT and JD prepared figures. JD, ZS-K, and DB participated in assembly and editing of the final manuscript.

## Conflict of Interest Statement

The authors declare that the research was conducted in the absence of any commercial or financial relationships that could be construed as a potential conflict of interest.
