# Supplementary material for: Regulation of Plant Microprocessor Function in Shaping microRNA Landscape
Source: Front Plant Sci. 2018 Jun 5;9:753. doi: 10.3389/fpls.2018.00753 (PMC5996484; doi:10.3389/fpls.2018.00753)
Supplement: Supplementary file 1 [file Table_1.PDF]

Supplementary Material

Regulation of plant Microprocessor function in shaping microRNA landscape

Dolata Jakub[1], Taube Michał[1], Bajczyk Mateusz[1] , Jarmołowski Artur[1], Szweykowska-Kulińska Zofia[1*], Bielewicz Dawid[1*]

[1]Department of Gene Expression, Institute of Molecular Biology and Biotechnology, Faculty of Biology, Adam Mickiewicz University in Poznan, Poznan, Poland.

*** Correspondence:**
Corresponding authors:
Prof. Zofia Szweykowska-Kulińska -zofszwey@amu.edu.pl
Dr. Dawid Bielewicz - bieda@amu.edu.pl

Supplementary Table S1. The list of the phosphorylation sites within the HYL1 and SERRATE proteins.

HYL1

Amino acid	Position in sequence	Domain	Reference
Predicted phosphorylation sites and functionally verified			
Serine	42*	dsRBD 1	Manavella et al. 2012
Serine	159*	dsRBD 2	Manavella et al. 2012
Experimentally detected phosphorylation sites			
Threonin	31	dsRBD 1	Durek et al. 2009
Serine	271**	C-terminal repeats domain	Manavella et al. 2012
Serine	299**	C-terminal repeats domain	Manavella et al. 2012
Serine	327**	C-terminal repeats domain	Manavella et al. 2012
Serine	411**	C-terminal repeats domain	Manavella et al. 2012

* Sites predicted by NetPhos 2.0 algorithm and confirmed via functional studies (Manavella et al. 2012).
* * The same sequence of the detected peptide (EAAFGSVETEK) is present in four repeats in C-terminal domain. It is not clear whether serine phosphorylation occurs in each of the four repeats or is present in one, two or three of them. (Manavella et al. 2012).

SERRATE (phosphorylation sites identified from phosphoproteomic data)

Amino acid	Position in sequence	Domain	Reference
Serine	20	N-terminal domain	Reiland et al. 2011, Wang et al. 2013, Wang et al. 2013b, Lin et al. 2015, Mattei et al. 2016

Threonin	21	N-terminal domain	Reiland et al. 2011, Wang et al. 2013, Lin et al. 2015, Mattei et al 2016
Serine	22	N-terminal domain	Reiland et al. 2011, Wang et al. 201b, 3 Rayapuram et al. 2014
Serine	23	N-terminal domain	Reiland et al. 2011, Wang et al. 2013b, Rayapuram et al. 2014
Serine	24	N-terminal domain	Mattei et al. 2016
Serine	31	N-terminal domain	Reiland et al. 2011, Wang et al. 2013
Serine	32	N-terminal domain	Reiland et al. 2011,
Serine	33	N-terminal domain	Reiland et al. 2011,
Serine	76	N-terminal domain	Reiland et al. 2009, Nakagami et al. 2010, Reiland et al. 2011
Serine	90	N-terminal domain	Sugiyama et al. 2008, Nakagami et al. 2010, Choudhary et al. 2015
Serine	92	N-terminal domain	Nakagami et al. 2010, Choudhary et al. 2015
Tyrosine	97	N-terminal domain	Sugiyama et al. 2008, Nakagami et al. 2010
Serine	291	N-terminal domain	Reiland et al. 2009, Reiland et al. 2011, Wang et al. 2013b
Threonin	294	Core domain -Middle segment	Reiland et al. 2009, Wang et al. 2013, Wang et al. 2013b
Serine	295	Core domain -Middle segment	Sugiyama et al. 2008, Reiland et al. 2009, Nakagami et al. 2010, Wang et al. 2013b, Roitinger et al. 2015, Bhaskara et al. 2017
Serine	299	Core domain -Middle segment	Reiland et al. 2009, Reiland et al. 2011, Nakagami et al. 2010, Wang et al. 2013, Wang et al. 2013b, Roitinger et al. 2015, Bhaskara et al. 2017
Serine	689	C-terminal domain	Hoehenwarter et. al. 2013, Zhang et al. 2013

Manavella PA, Hagmann J, Ott F, Laubinger S, Franz M, Macek B, Weigel D. Fast-forward genetics identifies plant CPL phosphatases as regulators of miRNA processing factor HYL1. Cell. 2012 Nov 9;151(4):859-70. doi: 10.1016/j.cell.2012.09.039. PubMed PMID: 23141542.

Durek P, Schmidt R, Heazlewood JL, Jones A, MacLean D, Nagel A, Kersten B, Schulze WX. PhosPhAt: the Arabidopsis thaliana phosphorylation site database. An update. Nucleic Acids Res. 2010 Jan;38(Database issue):D828-34. doi: 10.1093/nar/gkp810. Epub 2009 Oct 30. PubMed PMID: 19880383; PubMed Central PMCID: PMC2808987.

Mattei B, Spinelli F, Pontiggia D, De Lorenzo G. Comprehensive Analysis of the Membrane Phosphoproteome Regulated by Oligogalacturonides in Arabidopsis thaliana. Front Plant Sci. 2016 Aug 2;7:1107. doi: 10.3389/fpls.2016.01107. eCollection 2016. PubMed PMID: 27532006; PubMed Central PMCID: PMC4969306.

Wang X, Bian Y, Cheng K, Gu LF, Ye M, Zou H, Sun SS, He JX. A large-scale protein phosphorylation analysis reveals novel phosphorylation motifs and phosphoregulatory networks in Arabidopsis. J Proteomics. 2013 Jan 14;78:486-98. doi: 10.1016/j.jprot.2012.10.018. Epub 2012 Oct 27. PubMed PMID: 23111157.

Lin LL, Hsu CL, Hu CW, Ko SY, Hsieh HL, Huang HC, Juan HF. Integrating Phosphoproteomics and Bioinformatics to Study Brassinosteroid-Regulated Phosphorylation Dynamics in Arabidopsis. BMC Genomics. 2015 Jul 18;16:533. doi: 10.1186/s12864-015-1753-4. PubMed PMID: 26187819; PubMed Central PMCID: PMC4506601.

Wang P, Xue L, Batelli G, Lee S, Hou YJ, Van Oosten MJ, Zhang H, Tao WA, Zhu JK. Quantitative phosphoproteomics identifies SnRK2 protein kinase substrates and reveals the effectors of abscisic acid action. Proc Natl Acad Sci U S A. 2013 Jul 2;110(27):11205-10. doi: 10.1073/pnas.1308974110. Epub 2013 Jun 17. PubMed PMID: 23776212; PubMed Central PMCID: PMC3703982.

Reiland S, Finazzi G, Endler A, Willig A, Baerenfaller K, Grossmann J, Gerrits B, Rutishauser D, Gruissem W, Rochaix JD, Baginsky S. Comparative phosphoproteome profiling reveals a function of the STN8 kinase in fine-tuning of cyclic electron flow (CEF). Proc Natl Acad Sci U S A. 2011 Aug 2;108(31):12955-60. doi: 10.1073/pnas.1104734108. Epub 2011 Jul 18. PubMed PMID: 21768351; PubMed Central PMCID: PMC3150903.

Reiland S, Messerli G, Baerenfaller K, Gerrits B, Endler A, Grossmann J, Gruissem W, Baginsky S. Large-scale Arabidopsis phosphoproteome profiling reveals novel chloroplast kinase substrates and phosphorylation networks. Plant Physiol. 2009 Jun;150(2):889-903. doi: 10.1104/pp.109.138677. Epub 2009 Apr 17. PubMed PMID: 19376835; PubMed Central PMCID: PMC2689975.

Nakagami H, Sugiyama N, Mochida K, Daudi A, Yoshida Y, Toyoda T, Tomita M, Ishihama Y, Shirasu K. Large-scale comparative phosphoproteomics identifies conserved phosphorylation sites in plants. Plant Physiol. 2010 Jul;153(3):1161-74. doi: 10.1104/pp.110.157347. Epub 2010 May 13. PubMed PMID: 20466843; PubMed Central PMCID: PMC2899915.

Hoehenwarter W, Thomas M, Nukarinen E, Egelhofer V, Röhrig H, Weckwerth W, Conrath U, Beckers GJ. Identification of novel in vivo MAP kinase substrates in Arabidopsis thaliana through use of tandem metal oxide affinity chromatography. Mol Cell Proteomics. 2013 Feb;12(2):369-80. doi: 10.1074/mcp.M112.020560. Epub 2012 Nov 20. PubMed PMID: 23172892; PubMed Central PMCID: PMC3567860.

Zhang H, Zhou H, Berke L, Heck AJ, Mohammed S, Scheres B, Menke FL. Quantitative phosphoproteomics after auxin-stimulated lateral root induction identifies an SNX1 protein phosphorylation site required for growth. Mol Cell Proteomics. 2013 May;12(5):1158-69. doi: 10.1074/mcp.M112.021220. Epub 2013 Jan 17. PubMed PMID: 23328941; PubMed Central PMCID: PMC3650328.

Choudhary MK, Nomura Y, Wang L, Nakagami H, Somers DE. Quantitative Circadian Phosphoproteomic Analysis of Arabidopsis Reveals Extensive Clock Control of Key Components in Physiological, Metabolic, and Signaling Pathways. Mol Cell Proteomics. 2015 Aug;14(8):2243-60. doi: 10.1074/mcp.M114.047183. Epub 2015 Jun 19. PubMed PMID: 26091701; PubMed Central PMCID: PMC4528250.

Sugiyama N, Nakagami H, Mochida K, Daudi A, Tomita M, Shirasu K, Ishihama Y. Large-scale phosphorylation mapping reveals the extent of tyrosine phosphorylation in Arabidopsis. Mol Syst Biol. 2008;4:193. doi: 10.1038/msb.2008.32. Epub 2008 May 6. PubMed PMID: 18463617; PubMed Central PMCID: PMC2424297.

Roitinger E, Hofer M, Köcher T, Pichler P, Novatchkova M, Yang J, Schlögelhofer P, Mechtler K. Quantitative phosphoproteomics of the ataxia telangiectasia-mutated (ATM) and ataxia telangiectasia-mutated and rad3-related (ATR) dependent DNA damage response in Arabidopsis thaliana. Mol Cell Proteomics. 2015 Mar;14(3):556-71. doi: 10.1074/mcp.M114.040352. Epub 2015 Jan 5. PubMed PMID: 25561503; PubMed Central PMCID: PMC4349977.

Rayapuram N, Bonhomme L, Bigeard J, Haddadou K, Przybylski C, Hirt H, Pflieger D. Identification of novel PAMP-triggered phosphorylation and dephosphorylation events in Arabidopsis thaliana by quantitative phosphoproteomic analysis. J Proteome Res. 2014 Apr 4;13(4):2137-51. doi: 10.1021/pr401268v. Epub 2014 Mar 17. PubMed PMID: 24601666.

Bhaskara GB, Wen TN, Nguyen TT, Verslues PE. Protein Phosphatase 2Cs and Microtubule-Associated Stress Protein 1 Control Microtubule Stability, Plant Growth, and Drought Response. Plant Cell. 2017 Jan;29(1):169-191. doi: 10.1105/tpc.16.00847. Epub 2016 Dec 23. PubMed PMID: 28011693; PubMed Central PMCID: PMC5304354.
